# Brain metastases: Single-dose radiosurgery versus hypofractionated stereotactic radiotherapy: A retrospective study

**Published:** 2020-07-08

**Authors:** Carolina de la Pinta, E. Fernández-Lizarbe, D. Sevillano, A. B. Capúz, M. Martín, R. Hernanz, C. Vallejo, M. Martín, S. Sancho

**Affiliations:** ^1^Department of Radiation Oncology, Ramón y Cajal Hospital, Madrid, Spain; ^2^Department of Medical Physics, Ramón y Cajal Hospital, Madrid, Spain

**Keywords:** brain metastases, hypofractionated, radiosurgery, single fraction radiosurgery, stereotactic radiosurgery

## Abstract

**Background:**

Radiosurgery is employed for the treatment of brain metastases. The aim of this study is to evaluate the efficacy and tolerability of single-dose radiosurgery (SRS) compared to hypofractionated stereotactic radiotherapy (hFSRT).

**Materials and Methods:**

Between 2004 and 2018, we analyzed treatments of 97 patients with 135 brain metastases. Fifty-six patients were treated with SRS, and 41 patients were treated with hFSRT. Median dose was 16 Gy (12-20 Gy) for the SRS group and 30 Gy in 5-6 fractions for the hFSRT group. hFSRT was used for larger lesions and lesions located near critical structures. Kaplan-Meier curves were constructed for overall survival (OS) and local control (LC).

**Results:**

Median age was 64 years (range, 32-89 years). Median survival was 10 months (1-68 months). With a median follow-up of 10 months, no significant differences in OS between groups were found (*P*=0.21). LC for all patients was 67%. Local progression-free survival (LPFS) at 6 months and 1 year was 71% and 60% for the SRS group, respectively, and 80% and 69% for the hFSRT group, respectively (*P*=0.93). Although hFSRT was used for larger lesions and lesions in adverse locations, LPFS was not inferior compared to lesions treated with SRS. We observed acute toxicity grade 1-2 in 25 patients (25.8%). Late complications were observed in 11 patients (11.3%). Acute and late toxicity was similar in the SRS- and hFSRT-treated patients (*P*=0.63 and *P*=0.11, respectively). Brain recurrence occurred in 37.5% and 14.6% in the hFSRT and SRS group, respectively (*P*=0.06).

**Conclusions:**

Since patients treated with hFSRT exhibited similar survival and LPFS rates without differences in toxicity compared to those treated with SRS, hFSRT can be beneficial, particularly for patients with brain metastases.

**Relevance for Patients:**

Hypofractionated schemes in stereotactic radiosurgery offers treatment alternatives to patients with large lesions or lesions near critical structures.

## 1. Introduction

The most common intracranial malignancies in adults are brain metastases. Brain metastases are present in approximately 20-40% of cancer patients [1]. Treatment options include whole-brain radiation therapy (WBRT), surgery, and radiosurgery. The addition of WBRT to radiosurgery may improve local control (LC) but may increase the risk of side effects such as cognitive deficits. However, survival is similar with the use of radiosurgery combined with WBRT versus radiosurgery alone. The combination of surgery and WBRT is not superior to the combination of radiosurgery and WBRT [2-6]. Yamamoto *et al.*, in a prospective multicenter study, analyzed radiosurgery without WBRT in patients with >5 metastases and >10 metastases. They concluded that radiosurgery without WBRT could be an alternative treatment option in patients with 5-10 metastases without differences in LC from patients with 2-4 metastases [7].

Single-dose radiosurgery (SRS) is considerably less invasive than surgery. The risk of toxicity with SRS increases when tumors are large or are located near or within critical brain structures. In these cases, the use of fractionated radiotherapy may be beneficial to avoid severe toxicities [8]. The most common chronic side effect of SRS is brain radionecrosis (RN), which is associated with the presence of different neurological deficits in up to one-third of patients [9,10]. Hypofractionated stereotactic radiotherapy (hFSRT) has been used as an alternative to SRS with high LC rates. Using doses of 24-35 Gy in 3 to 5 fractions, several retrospective studies have reported an LC rate of 70-90%/year, with a variable risk of RN between 2% and 15% [11,12].

The aim of this study is to compare efficacy and tolerability of two different schemes of intracranial radiotherapy: SRS and hFSRT. Our hypothesis is that radiosurgery is better in local progression (LP)-free survival (LPFS) and has lower rates of side effects than hFSRT.

## 2. Patients and Methods

### 2.1. Patient characteristics

We retrospectively analyzed 97 patients with 135 metastases who were treated with SRS or hFSRT, between January 2004 and December 2018.

Median age was 64 years (range, 32-89 years). About 53.6% of the study cohort was comprised of males. Karnofsky Performance Status (KPS) was more than 70 in 72.2% of patients. Primary tumors were: Lung in 68%, breast in 14.4%, renal in 4.1%, and other tumors (gastrointestinal or melanoma) in 13.5%. In 42.7% (41 patients), the primary tumor was active and 34.7% (33 patients) had other metastases. Predominant histology was adenocarcinoma in 59.8% of patients. About 67% of patients had only one brain metastases, 19.6% had two brain metastases, 6.2% had three brain metastases, 4.1% had four brain metastases, and 3% had more than four brain metastases. The characteristics of the patients are listed in Table 1.

**Table 1:** Clinical characteristics of the 97 patients (with 137 brain metastases) treated with RS for brain metastases

Clinical characteristics	Patients who received SRS *n*=56	Patients who received hFSRT *n*=41
Age (y)		
Median	63	64
Range	32-87	32-89
Gender (female/male)	24/32	21/20
Histology		
NSCLC	43	23
Breast	5	9
Melanoma	1	0
RCC	1	3
GI	3	1
Other	3	5
KPS		
70-100	42	29
<70	14	12
Extracranial disease		
Present	31	28
Absent	25	13
Number of metastases		
1	35	30
2	14	5
3	3	3
≥4	4	3
Previous WBRT		
Yes	26	12
No	30	29

SRS: Stereotactic radiosurgery, hFSRT: Hypofractionated stereotactic radiotherapy, NSCLC: Non-small cell lung cancer, KPS: Karfsnosky Performance Status, WBRT: Wholebrain radiotherapy, RCC: Renal cell carcinoma, GI: Gastrointestinal

Fifty-six patients (54.3%) had been treated with SRS and 41 (45.7%) had been treated with hFSRT. Median SRS dose was 16 Gy (12-20 Gy) and hFSRT dose was 30 Gy in 5 or 6 fractions. hFSRT was recommended in large-sized tumors, irregular shaped tumors, or in tumors located near or within a critical structure. In this study, 38 patients (39.1%) had received WBRT before SRS or hFSRT and 28 patients (28.4%) had previously undergone surgery, showing unbalanced groups. No differences in the distribution of gender (*P*=0.38) or the presence of extracranial metastases (*P*=0.36) were observed, but differences were observed in previous WBRT history (26 patients and 12 patients in SRS and hFSRT-group, respectively, *P*=0.036) between the SRS and hFSRT groups.

hFSRT was employed when tumor characteristics did not permit to accomplish dose constraints for critical structures. Volume of healthy brain tissue receiving doses larger than 10 or 12 Gy in a single fraction was the main reason for switching to an hFSRT treatment option. Irregular tumors, as well as the proximity of critical structures that could not be easily spared with a cone-based technique, were secondary reasons for fractionating the treatment.

### 2.2. Treatment characteristics with SRS or hFSRT

For immobilization, a tight thermoplastic mask was applied. Computed tomography images with a slice thickness of 1 or 2 mm were obtained, and these computed tomography images were fused with magnetic resonance imaging (MRI) using an image fusion software. The gross tumor volume (GTV) was defined as the contrast-enhancing tumor. The planning target volume was defined by adding a 3 mm margin to the GTV. We applied 3 mm margin due to our linac limitations, it was not a dedicated facility for radiosurgery, so we aimed to eliminate any possible uncertainty. Dose-limiting structures were eyes, lens, optic nerves and chiasm, brain, brainstem, and cochlea, so they were contoured in all patients.

**Figure 1:**
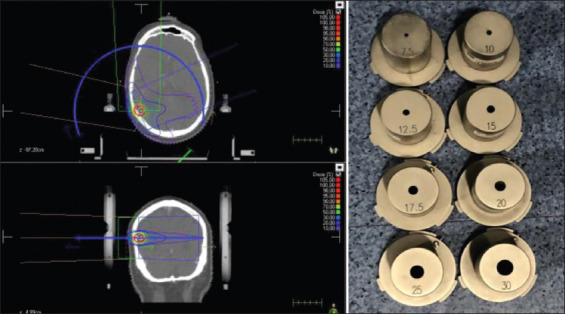
Cone-based stereotactic radiosurgery treatment and different cones diameters.

**Figure 2:**
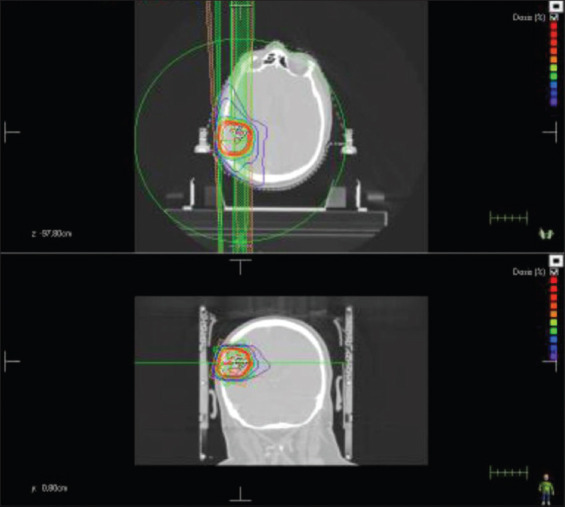
Hypofractionated stereotactic radiotherapy treatment with volumetric modulated arc therapy.

hFSRT was administered every day or every 2 days for 1.5-2 weeks, working days only. A cone-based treatment delivery technique with multiple non-coplanar arcs was used in the most of cases of SRS treatments (rest of cases with Intensity Modulated Radiotherapy [IMRT]), while hFSRT treatments were delivered using 3D, IMRT, or Volumetric Modulated Arc Therapy (VMAT) with both coplanar and non-coplanar beam settings, depending on the year of treatment. When V12 Gy of the normal brain was higher than 10 cc, there were two options: SRS at lower doses, or a new intervention with hFSRT. The decision was physician-dependent. V12 Gy was a strict constraint (Figures 1 and 2).

### 2.3. Follow-up and statistics

The endpoints were overall survival (OS) and LPFS. After treatment, patients were followed up with serial neurologic and radiologic examinations. Follow-up MRI were obtained routinely 1 month after treatment, followed by 3-month intervals, or in the event of unexpected neurologic deterioration or progression. Response criteria were defined as RECIST: Complete response (CR); partial response (PR), at least 50% decrease; progressive disease (PD), at least 25% increase; and stable disease (SD), neither PR nor PD. LP was defined as progression within the treatment volume. Regional progression (RP) was defined as intracranial progression outside of the treatment volume. Toxicity was recorded according to the National Cancer Institute Common Terminology Criteria for Adverse Events (version 4.0). Differences between the groups were evaluated using Student’s test. Survival rates were calculated from the date of the start of treatment to the date of occurrence of an event or the date of the last follow-up by use of the Kaplan-Meier method and examined for significance using the log-rank and generalized Wilcoxon test. All analyses employed the conventional *P*<0.05 level of significance. SPSS statistical software (version 21.0; SPSS, Chicago, IL) was used for statistical analyses.

## 3. Results

### 3.1. Tumor response and patterns of failure

Tumor response was evaluated in 91 patients with 131 lesions after the exclusion of 6 patients for whom no follow-up images were available. The CR, PR, SD, and PD rates were 46.5%, 25.6%, 6.9%, and 21.0%, respectively, in the SRS group and 56.2%, 31.2%, 6.3%, and 6.3%, respectively, in the hFSRT group, with no statistically significant differences between groups (*P*=0.5).

### 3.2. LP–free survival (LPFS) and RP –free survival

**Figure 3:**
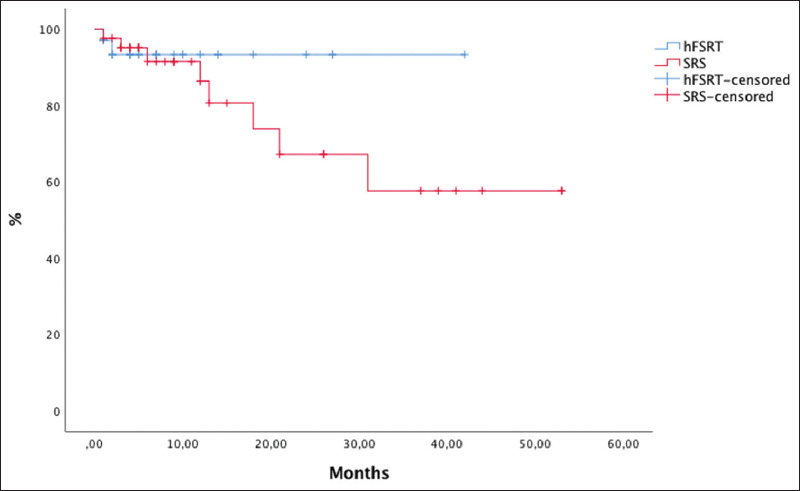
Kaplan-Meier curves with comparison local progression-free survival between stereotactic radiosurgery and hypofractionated stereotactic radiotherapy.

LPFS at 6 months and 1 year was 71% and 60%, respectively, for the SRS group and 80% and 69%, respectively, for the hFSRT group (*P*=0.93) (Figure 3). Although hFSRT was used for larger lesions or lesions located in precarious locations, LPFS was not inferior compared to SRS. Intracranial progression occurred in 37.5% and 14.6% of the cases in the hFSRT group and the SRS group, respectively (*P*=0.06).

### 3.3. OS

**Figure 4:**
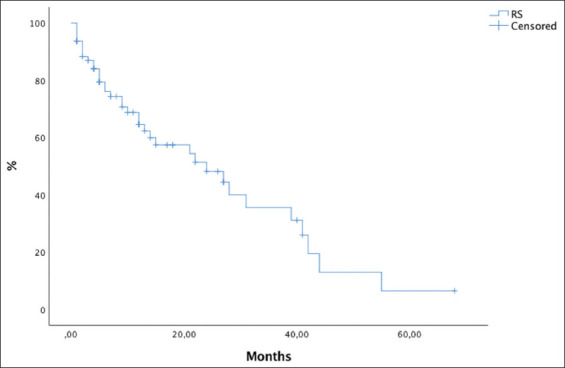
Kaplan-Meier curves with global overall survival.

**Figure 5:**
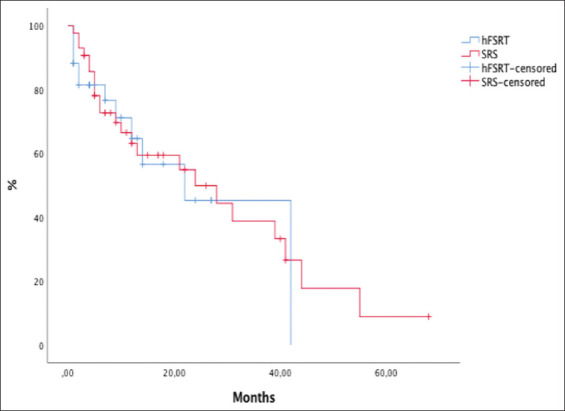
Kaplan-Meier curves comparing overall survival (OS) for stereotactic radiosurgery to OS for hypofractionated stereotactic radiotherapy.

Thirty patients had died at the last follow-up. With a median follow-up of 10 months (range, 1-68 months), 16.3 months for the SRS group and 9.2 months for the hFSRT group (*P*=0.025), OS rates at 6 months and 1 year were 70% and 63% for the whole group. OS rates were 70% and 62% for the SRS group, and 81% and 70% for the hFSRT group (*P*=0.85). Survival outcome in the hFSRT group was not inferior to that of the SRS group (Figures 4 and 5).

### 3.4. Cause of death

Of 30 patients, 20 (66.7%) had died of PD within the central nervous system, (20.0%) patients had died of systemic tumor progression, and 4 patients (13.3%) had died of other diseases.

### 3.5. Toxicity

All patients with SRS received corticosteroids after treatment. In the hFSRT group, corticosteroids were administered only if patients had edema with mass effect. We classified acute or late toxicity according to the Common Terminology Criteria for Adverse Events, version 4.0. Mild symptoms without indicated intervention were classified as grade 1; moderate symptoms requiring non-invasive intervention, such as drug administration, as grade 2; and severe symptoms requiring hospitalization were classified as grade 3. The acute toxicities observed in our series were grade 1 and 2, including brain edema (22 patients) and seizure without sequels (3 patients). Latent toxicity consisted of RN (3 patients), post-radiation syndrome (1 patient), long-term seizures (5 patients), pan-hypopituitarism (1 patient), and diabetes insipidus (1 patient). Acute toxicity was similar in both groups (29% vs. 23%, *P*=0.63) and chronic toxicity was more frequent in the hFSRT group than in the SRS group (19.5% vs. 7.1%, *P*=0.11).

We divided the SRS group into two subgroups: With dose reduction (<16 Gy) and without dose reduction (>16 Gy). We analyzed differences in tumor response, PFS, and toxicity between these two subgroups. We found no statistically significant differences in tumor response (*P*=0.24), PFS (*P*=0.15), or toxicity (acute: *P*=0.21; late: *P*=0.63) between subgroups. We also analyzed whether there were differences in the treatment with WBRT. In the SRS subgroup with dose reduction (23 patients), 16 patients (61.6%) had received WBRT compared to the SRS subgroup without dose reduction (33 patients), where only 10 patients had received WBRT (38.4%) (*P*=0.002).

## 4. Discussion

SRS is an effective treatment for brain metastases. However, SRS is not always possible, especially in large tumors and in tumors located near critical organs where doses are limited by the risk of increased side effects. In these cases, an alternative is the use of hFSRT. In our center, we perform radiosurgery with cone collimators in most of the cases, and occasionally we switch to hFSRT with VMAT or IMRT for irregular metastases. In our study, we analyzed differences between SRS and hFSRT in LC, toxicity, and survival in a single-center cohort.

### 4.1. Doses and LC

As doses in hFSRT have not been established, it is very difficult to compare results from different series. Most studies comparing doses use the Biologically Effective Dose (BED). However, the reliability of the linear-square (LQ) model for hypofractionated radiotherapy has been questioned. Marcrom compared 25 Gy and 30 Gy in 5 fractions; 30 Gy was associated with a better LC (72% vs. 40%) [13]. Murai *et al*. [14] conducted a study comparing 3 dose levels: Level I, 18-22 Gy in 3 fractions or 21-25 Gy in 5 fractions; level II, 22-27 Gy in 3 fractions or 25-31 Gy in 5 fractions; and level III, 27-30 Gy in 3 fractions or 31-35 Gy in 5 fractions. The authors concluded that the highest dose levels 27-30 Gy in 3 fractions and 31-35 Gy in 5 fractions were tolerable and effective in controlling large brain metastases. At our institution, we use 30 Gy in 5-6 fractions (BED: 43.3-47.7 Gy) for the hFSRT and we use 15-20 Gy in 1 fraction (BED: 28.6-41.0 Gy).

In terms of LC, SRS studies report variable results, with 1-year LPFS between 68 and 81% [1,8] and LP in 3-13% of the cases. In our study, LP was 21% for the whole group. This difference could be explained by the low median doses (16 Gy) of SRS in our institution compared to other studies with median doses up to 20 Gy [1,8]. Furthermore, this is in relation to studies such as that of Feuvret *et al*. [15] that used 14 Gy in 1 fraction for metastases >3 cm with an LC of 58%.

Different studies in hFSRT had reported that the LPFS at 1 year was between 68% and 76% [16-18] and that LP was 19-60% [5,13]. LP in our series was only 6.3%. For the hFSRT, we used 30 Gy in 5-6 fractions, similar to other studies [1]. The use of BED higher doses, as in the study by Minniti *et al*. [8] with 27 Gy in 3 fractions, showed an LC of 91%, similar to our group. The studies are summarized in Table 2.

**Table 2:** Studies that compare single-dose SRS and hFSRT in brain metastases

Studies	n	Dose/fr	WBRT	LC	Overall Survival	Toxicity
Kim *et al*. 2011 [1]	Single-dose SRS: 58 patients (81 lesions) hFSRT: 40 patients (49 lesions)	20 Gy/1 fr 36 Gy/6 fr	Single-dose SRS: 12 patients hFSRT 16 patients	16% CR 7% PD 15% CR 0% PD	MS 16	17% single-dose SRS 5% hFSRT *P*=0.05
Minniti * et al*. 2016 [8]	289 patients 343 lesions y>2cm	18 Gy/1 fr o 15-16 Gy si >3 cm 27 Gy/3 fr	5 patients	Single-dose SRS: 77% hFSRT: 91%	1-y 58% 2-y 24%	20% single-dose SRS RN 8% hFSRT RN
Feuvret [15]	24 patients single-dose SRS 12 patients hFSRT	14 Gy/1 fr >3 cm 23.1 Gy/3 fr	-	58% 100%	MS 5.5 m MS 16.8 m	6 patients G1-2 2 patients G2
Donovan *et al.* 2019 [27]	90 patients	24 Gy/1 fr 21 Gy/3 fr	12 patients	Recurrence 8 patients 6 patients single-dose SRS 2 patients hFSRT	MS 11.7 m	16p RN (21/62 lesions; 4 symptomatic RN) 10 patients 1 fr 11 patients 3fr
Present study	97 patients (135 lesions) 56 patients Single-dose SRS 41 patients hFSRT	I: 12-15 Gy/1 fr II: 16-20 Gy/1 fr III: 30 Gy/5-6 fr	16 patients 10 patients 12 patients	82.6% 78.2% 93.7%	MS 10 m	Single-dose: Acute grade 1-2: 29% Late: 7.1% hFSRT: Acute grade 1-2: 23% Late: 19.5%

SRS: Stereotactic radiosurgery, hFSRT: Hypofractionated stereotactic radiotherapy, WBRT: Whole-brain radiotherapy, CR: Complete response, PD: Progression disease, MS: Median survival, RN: Radionecrosis

Wiggenraad *et al*. [19], in their systematic review, reported a 6-month LC of 80% in almost all series, independently of the dose prescribed. The 20-month control was 80%, 60%, and 50% for >21 Gy, >18 Gy, and <15 Gy, and 70% for hFSRT. A BED of >40 Gy was associated with a 20-month LC rate of ≥70%. In our institution, the LC at a median of follow-up of 10 months was 69% in the SRS group (12-20 Gy) and 93.7% for the hFSRT group.

To provide good LC, suitable doses in SRS appear to be ≥18-20 Gy and in hFSRT a total BED of at least 50 Gy (500 cGy per fraction in 6-7 fractions).

### 4.2. OS

Several studies have identified factors influencing OS, including the general condition of patients, that were measured with scales such as Recursive Partitioning Analysis, Graded Prognostic Assessment, or Performance Status [13,20,21]. The clinical situation of the patient, the presence or absence of extracranial metastases, the size of the brain metastases, and the histology or the number of metastases are the most important prognostic factors [22].

In most series, 30-50% of patients were alive after 12 months. Minniti *et al*. showed 1- and 2-year survival rates of 58% and 24%, respectively. In our series, the 1-year survival rate was 63% for all patients and 62% and 70% in the SRS group and the hFSRT group, respectively. These data are similar to what has been reported in the literature.

### 4.3. Toxicity

Different authors have studied the radiobiological advantages of hypofractionation as a means to achieve adequate tumor control at limited side effects.

Dose limitations to brain parenchyma are difficult to compare due to the variability of doses and fractions and the toxicity criteria. In a series comparing SRS and hFSRT, Inoue *et al*. explained that V14 Gy may be predictive of RN, limited at V14 Gy for >7 cc [23]. The risk of RN can be maintained at <2-15% when using a BED of 90-127 Gy3 (a/b=3) (24-35Gy in 3-5 fractions). Different studies have shown that V12 Gy and V18 Gy are predictive factors for RN [24]. In our series, we limited V12 Gy for <10 cc in SRS and V4 Gy per fraction for <20 cc in hFSRT and V21 Gy for <20.9 cc and V28.8 Gy for <7 cc at ≥6 Gy per fraction.

Brain edema is the most frequent form of toxicity. In the study by Fahrig *et al*. [25], edema was present in 10% of patients, being more frequent after shorter courses than after longer courses of treatment. In our study, brain edema had manifested in 22 patients (22.7%); 11 patients in the SRS group and 11 patients in the hFSRT group.

RN is secondary to damage to brain cells, occurring between 6 months and 2 years after treatment. Several studies analyze its relationship with different variables such as dose, volume of the tumor, volume of normal brain irradiated, number of fractions, and combination with systemic treatments. In our study, we only analyzed symptomatic RN. RN was developed in 3 patients (3%); 2 patients in the hFSRT group; and 1 patient in the SRS group. RN is variable throughout studies, the incidence range is 8-34% [25-27]. Minniti *et al*. [8] observed 20% versus 8% of RN at SRS and hFSRT, respectively (*P*=0.004). Fahrig *et al*. [25] found differences in hFSRT schemes and incidence in RN. The authors found an RN rate of 22% in 5-6 fractions compared to 7% in 7 fractions. In the study by Zhuang *et al*. [28], it was observed higher rates of RN occur in 1-4 fractions compared to 5-8 fractions, with an overall risk of 11.5%. In our analysis, the effect of fractionation on RN was inconclusive but, although we showed no difference in toxicity between the SRS and hFSRT groups, previously published series seemed to show higher rates of RN following SRS. It is possible that we have not shown an association between RN and the maximum SRS dose in our patients due to the low rates of RN and because we only analyzed symptomatic RN. Other studies have shown that hFSRT may have a protective effect, reducing the risk of RN in large lesions. The overall risk of RN appears to be higher than expected when multiple treatments are administered simultaneously [24].

Our study has a number of limitations, including the fact that it is a retrospective study, the small sample size, and heterogeneity of the characteristics of patients. The definition of LC is not uniform among studies and its comparison is complicated. The diagnosis of radiographic RN is controversial, and we only evaluated symptomatic toxicity. Studies have demonstrated the limitations of LC for hFSRT. Finally, due to the volume of lesions treated, it may have influenced the prescribed dose and fractionation, making it difficult to perform an independent examination of the effect of fractionation in the patients in this study.

## 5. Conclusions

Despite the fact that adverse locations and larger volumes of brain metastases were more frequently observed in the hFSRT-group, patients treated with hFSRT exhibited similar survival and LPFS rates with similar toxicity in comparison to those treated with SRS. hFSRT treatment can particularly be beneficial in these patients. However, the optimal dose fractionation is still unknown and requires further investigation, but we consider 30-35 Gy in 6-7 fractions for hFSRT and 18-20 Gy for SRS as optimal options.
